# Bronchial tuberculosis with recurrent spontaneous pneumothorax: A case report

**DOI:** 10.1186/s12890-023-02374-y

**Published:** 2023-03-21

**Authors:** Ting Li, Yu-hong Li, Ming Zhang

**Affiliations:** 1 The Affiliated Hospital of Qinghai University, Xining, 810001 China; 2 Department of Respiratory Medicine, The Affiliated Hospital of Qinghai University, Xining, 810001 China

**Keywords:** Bronchial tuberculosis, Spontaneous pneumothorax, Secondary pneumothorax

## Abstract

**Background:**

Spontaneous pneumothorax associated with tuberculosis due to clinical manifestations, imaging findings and negative pleural biopsy is rare.

**Case report:**

A 43-year-old young woman went to the hospital several times because of recurrent dyspnea and was diagnosed with a right spontaneous pneumothorax. She underwent multiple closed thoracic drainage procedures, but the pneumothorax was not completely resolved. Pleural biopsy pathology was chronic inflammation; there was no evidence of tuberculosis. A small amount of pneumothorax persisted, intermittent dyspnea became more severe, and pneumothorax increased. Bronchoscopy showed thickening of the left lung lingular segment mucosa, and the bronchial lavage fluid gene X-PERT/rifampicin resistance test was positive. After one month of anti-tuberculosis treatment, the symptoms of short breath were completely relieved, and chest computerized tomography (CT) showed complete resolution of the right pneumothorax.

**Conclusions:**

When searching for the cause of spontaneous pneumothorax, people should not overlook tuberculosis-related secondary pneumothorax, which should be diagnosed and treated as soon as possible.

## Background

A pneumothorax is a collection of air outside the lung but within the pleural cavity [1]. Pneumothorax is divided into spontaneous pneumothorax and non-spontaneous (traumatic) pneumothorax. Among them, spontaneous pneumothorax is divided into primary spontaneous pneumothorax (PSP) and secondary spontaneous pneumothorax (SSP). Iatrogenic and non-iatrogenic pneumothorax are the two types of non-spontaneous pneumothorax. This article focuses on SSP. SSP is considered to be one of the causes of spontaneous pneumothorax [2]. The most common risk factors were chronic obstructive pulmonary disease (COPD), asthma, human immunodeficiency virus (HIV) combined with pneumocystis pneumonia, necrotizing pneumonia, tuberculosis nodules, and cystic fibrosis, followed by rare diseases. Based on the above, secondary spontaneous pneumothorax, as a potentially life-threatening disease, requires immediate action [3].

Case Report.

**Fig. 1:**
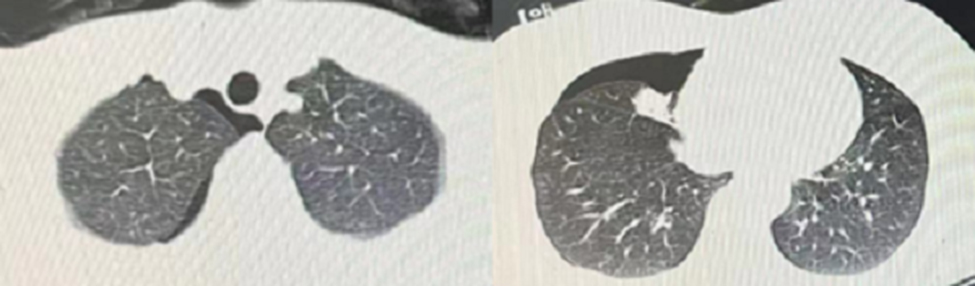
Right spontaneous pneumothorax April 2021 Provincial People ‘s Hospital

**Fig. 2:**
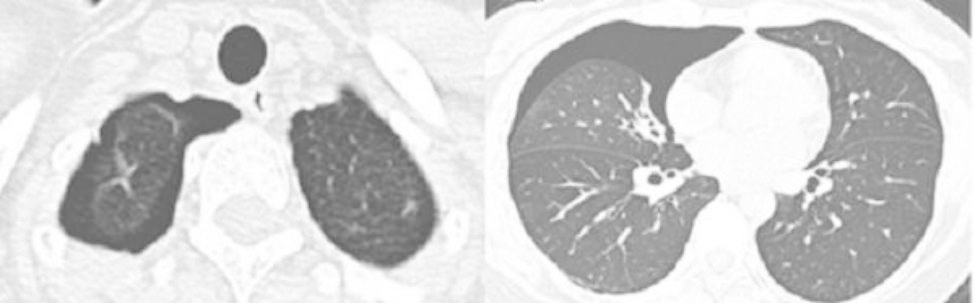
Right spontaneous pneumothorax January 2021 Our Hospital

**Fig. 3:**
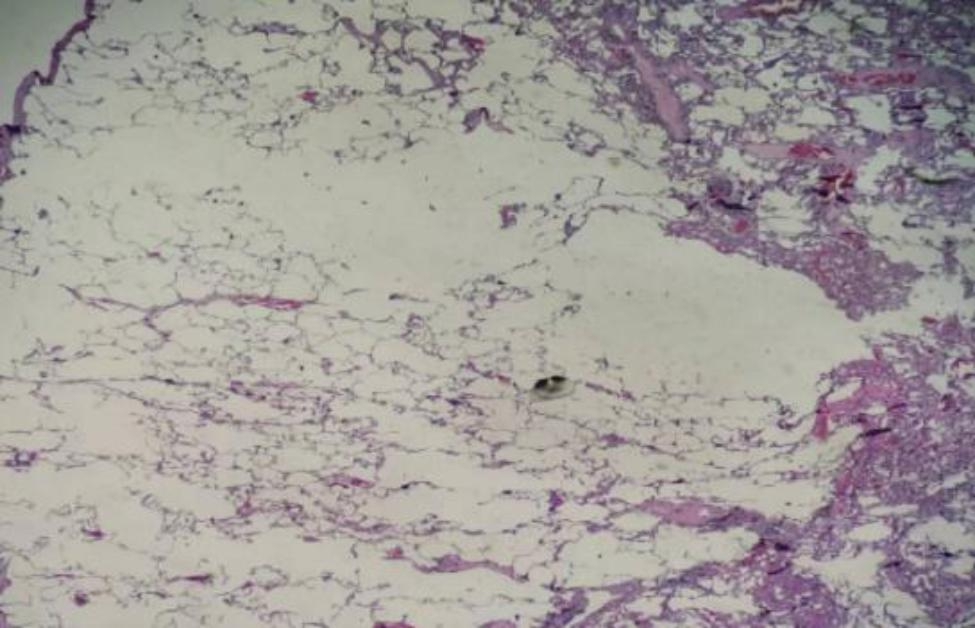
Postoperative pathological chronic inflammation, subpleural alveolar structural deformation

**Fig. 4:**
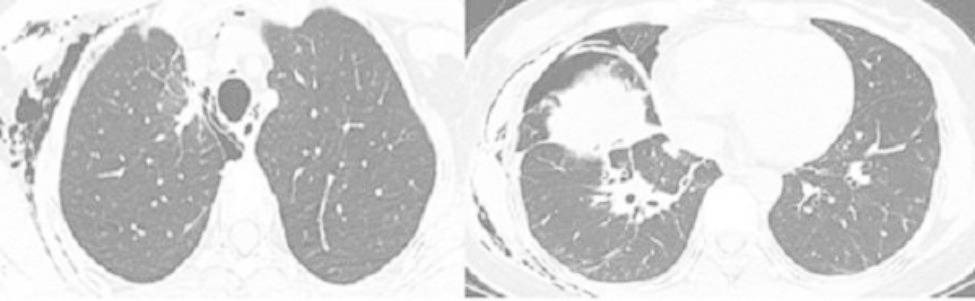
Small residual gas and subcutaneous emphysema in posterior right thoracic cavity

**Fig. 5:**
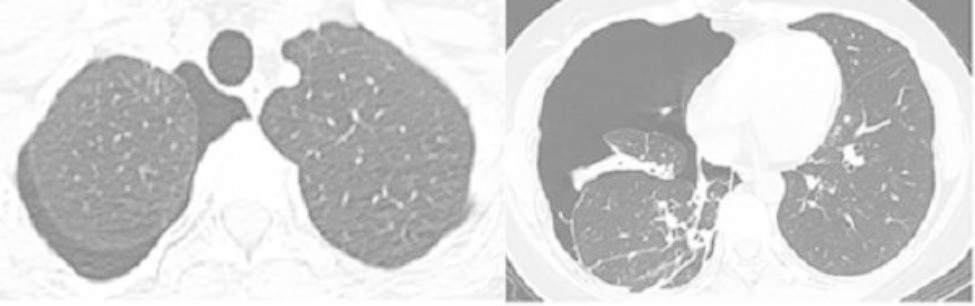
Right spontaneous pneumothorax July 2022

**Fig. 6:**
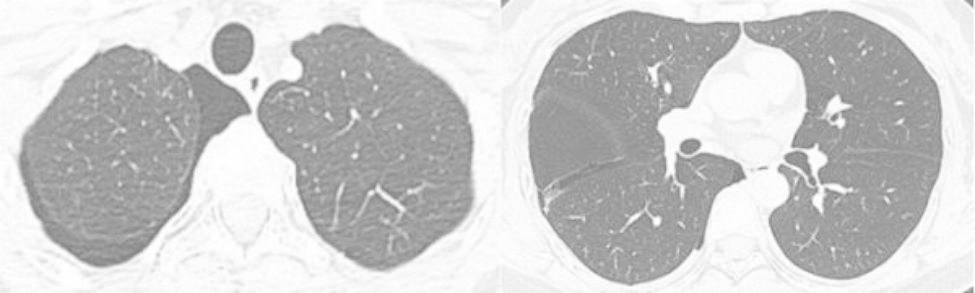
Admitted to our department due to dyspnea, spontaneous pneumothorax August 2022

**Fig. 7:**
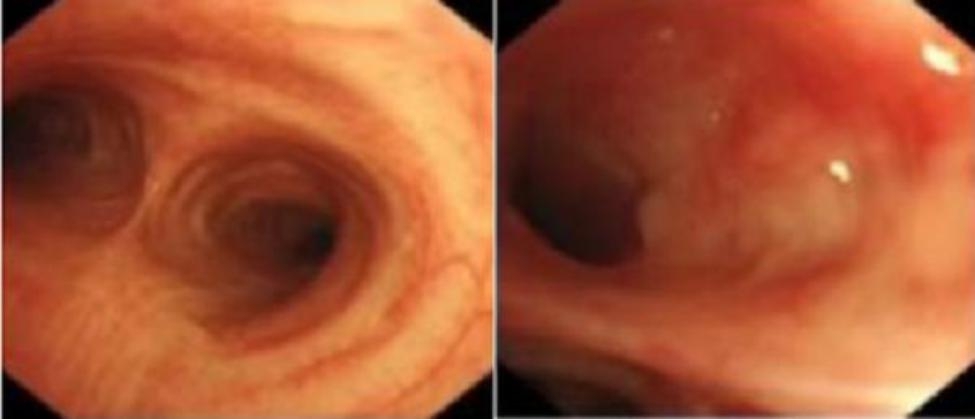
Bronchoscopy revealed local thickening of the mucosa of the lateral lingular branch of the left upper lobe

**Fig. 8:**
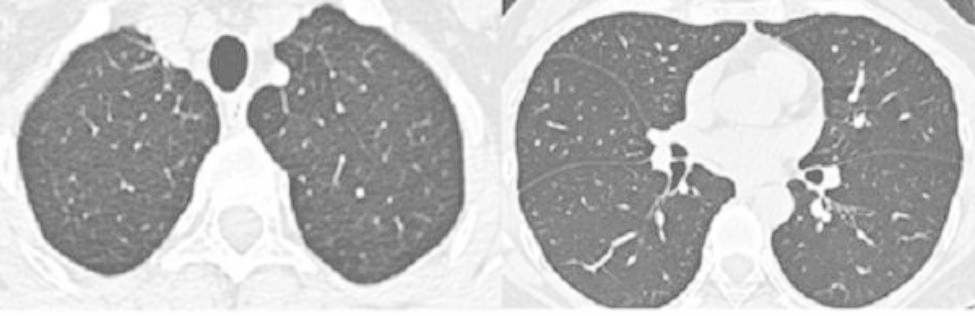
After 1 month of anti-tuberculosis treatment, the patient ‘s shortness of breath was completely relieved, chest CT was reviewed, and the right pneumothorax was completely resolved

A 43-year-old female patient was admitted in August 2022 because of repeated chest tightness and dyspnea for more than one year. In April 2021, the patient was diagnosed with a right spontaneous pneumothorax (Fig. 1) in the local provincial people ‘s hospital due to sudden chest tightness and dyspnea. The patient was treated with closed thoracic drainage. The pneumothorax was not completely resolved after the operation. Six months after the operation, the chest CT was reviewed. There was still a small amount of pneumothorax, and there was no obvious abnormality in the remaining lungs. In July 2021, the patient underwent a pleural biopsy without evidence of tuberculosis, caseous necrosis, or granulomatous lesions. On January 31,2022, chest tightness and dyspnea recurred. She was admitted to the Department of Thoracic Surgery of our hospital due to a right spontaneous pneumothorax with a large amount of pneumothorax (Fig. 2) and underwent thoracoscopy pleurodesis. The postoperative pathology was chronic inflammation, and alveolar structural deformation was seen under the pleura (Fig. 3). There was a small amount of residual gas and subcutaneous emphysema in the right thoracic cavity after the operation (Fig. 4), and the pneumothorax was still not completely resolved. On July 13,2022, closed thoracic drainage was performed again in thoracic surgery due to a right spontaneous pneumothorax (Fig. 5). In August 2022, due to breathing difficulties, a spontaneous pneumothorax was admitted to our department (Fig. 6). Admission examination vital signs are stable; oxygen saturation (SpO_2_): 89%, the right lung breath sound is reduced. A chest CT on the right side revealed a small amount of pneumothorax, symptoms gradually improved after being given rest and oxygen. Improve inflammation indicators, blood routine, urine routine, biochemical, erythrocyte sedimentation rate, and C-reaction protein (CRP) levels that are normal, and immune autoantibodies (ANA 1: 100). The rest are normal. In bronchoscopy, we found local thickening of the mucosa of the lateral branch of the lingular segment of the left upper lobe of the lung (Fig. 7), and no stenosis or new organisms were found in the remaining bronchi. Bronchoalveolar lavage fluid bacteria, fungal and mycobacterium tuberculosis culture negative, pathology for a small number of phagocytes, bronchial lavage fluid gene X-PERT / rifampicin resistance test positive, and rifampicin sensitive. After one month of anti-tuberculosis treatment with the HRZE (H: isoniazid, R: rifampicin, Z: pyrazinamide, E: ethambutol) regimen, the patient’s shortness of breath was completely relieved, and a chest CT showed complete resolution of the right pneumothorax. (Fig. 8).

Discussion and Conclusions.

Spontaneous pneumothorax is usually divided into primary and secondary and occurs in patients with chest trauma or lung potential underlying diseases [4]. Spontaneous pneumothorax can be secondary to a variety of lung diseases. Such as COPD, tuberculosis, etc. Patients with secondary pulmonary tuberculosis may have local fibrosis and contraction of the residual lung, and pneumothorax may occur after secondary bullae rupture [5]. It has been reported that the estimated incidence of spontaneous pneumothorax associated with active tuberculosis is only about 1–2% [6].

The cases we reported had no history of pulmonary tuberculosis and no symptoms of tuberculosis poisoning. Spontaneous pneumothorax occurred repeatedly. There was no evidence of tuberculosis in the pleural biopsy, and no caseous necrosis or granulomatous lesions. After one year, the pneumothorax increased, the bronchoalveolar lavage fluid was found to be positive for gene X-PERT, and the pneumothorax was completely resolved after anti-tuberculosis treatment.

After consulting the domestic and foreign literature in the past 10 years, no literature reported that the cause of pneumothorax without lung parenchymal lesions was screened for tuberculosis infection. The author hypothesizes the following reasons: (1) Pleural tuberculosis is mainly pleural inflammation caused by a high-intensity allergic immune response caused by Mycobacterium tuberculosis invading the pleural cavity. A pleural immune response may occur earlier than lung lesions. (2) The main feature of pulmonary tuberculosis is the destruction of lung parenchyma and loss of matrix metalloproteinases [7]. Pleural matrix metalloproteinases also appear prone to leak-like damage. (3) Latent tuberculosis infection is caused by a complex antigen of Mycobacterium tuberculosis that adapts itself. This antigen can persist for decades at inactive stages and has no evidence of clinically active tuberculosis [8]. Considering that this patient does not have the risk factors for PSP (such as smoking, a tall and thin body, pregnancy, Marfan syndrome, familial pneumothorax, etc.), and PSP occurs mostly in people between 20 and 30 years old. In the United States, the incidence rate is 7 cases per 100,000 people per year for men and 1 case per 100,000 people per year for women [1]. Therefore, PSP is temporarily excluded, and it cannot be diagnosed as PSP before the risk factors of SSP are excluded.

In short, in patients with recurrent spontaneous pneumothorax, tuberculosis-related spontaneous pneumothorax cannot be ignored. Risk factors for SSP should be investigated in good time, and spontaneous pneumothorax should be diagnosed and treated early.

## Data Availability

The datasets used and/or analyzed during the current study available from the corresponding author on reasonable request.

## Abbreviations

CT: Computerized tomography
PSP: Primary spontaneous pneumothorax
SSP: Secondary spontaneous pneumothorax
COPD: Chronic obstructive pulmonary disease
HIV: Human immunodeficiency virus
SpO_2_: Oxygen saturation
CRP: C-reaction protein
HRZE: H:isoniazid, R:rifampicin, Z:pyrazinamide, E:ethambutol.
