# Correction to: Bronchial tuberculosis with recurrent spontaneous pneumothorax: A case report

**DOI:** 10.1186/s12890-023-02461-0

**Published:** 2023-06-12

**Authors:** Ting Li, Yu-hong Li, Ming Zhang

**Affiliations:** 1 The Affiliated Hospital of Qinghai University, Xining, 810001 China; 2 Department of Respiratory Medicine, The Affiliated Hospital of Qinghai University, Xining, 810001 China

**Correction to: BMC Pulmonary Medicine (2023) 23:93**

10.1186/s12890-023-02374-y

Following publication of the original article, the authors flagged an error in Fig. 1: the figure had been erroneously replaced by a duplicate of Fig. 7. The article has since been updated to correct the figure. The authors thank you for reading and apologize for any inconvenience caused.

**Fig. 1:**
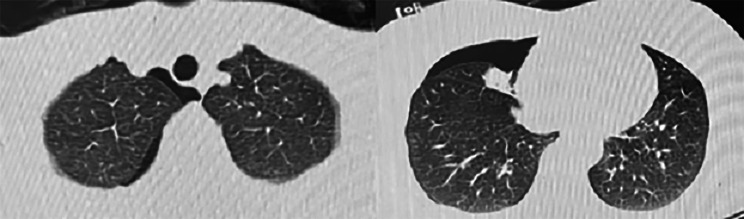
Right spontaneous pneumothorax April 2021 Provincial People’s Hospital

